# From Molecules to Models: miRNAs and Advanced Human Platforms of Neurodegeneration and Repair in Multiple Sclerosis

**DOI:** 10.3390/ijms26178740

**Published:** 2025-09-08

**Authors:** María Muñoz-San Martín, Lucía de la Guerra-Sasián, Gabriel Gárate, Jorge Madera, Andrea González-Suárez, Nadia C. Cavada-Bustamante, Vicente González-Quintanilla, Jennifer K. Dowling

**Affiliations:** 1 Brain Inflammation Group Ireland (BIGie), School of Pharmacy and Biomolecular Sciences, Royal College of Surgeons in Ireland, 123 St. Stephen’s Green, D02 YN77 Dublin, Ireland; 2 Instituto de Investigación Marqués de Valdecilla (IDIVAL), Hospital Universitario Marqués de Valdecilla & Universidad de Cantabria, Avda. Cardenal Herrera Oria s/n, 39011 Santander, Spain; 3 FutureNeuro, SFI Research Centre for Chronic and Rare Neurological Diseases, Royal College of Surgeons in Ireland, 123 St. Stephen’s Green, D02 YN77 Dublin, Ireland

**Keywords:** multiple sclerosis, miRNAs, neurorepair, remyelination, human models, iPSC, organoids

## Abstract

Beyond the potential role of microRNAs (miRNAs) as biomarkers, their participation in different biological and pathological processes observed in multiple sclerosis (MS) such as neuroinflammation, neurodegeneration and remyelination, makes them suitable candidates for therapeutic applications in neurorepair. Most studies addressing this reparative approach have been carried out using in vitro or in vivo model systems. However, functional differences between murine and human cells within the central nervous system (CNS) have been described, and certain mechanisms are distinctive in humans. The development of human models to investigate therapeutic interventions in neurological conditions including MS should be a priority to avoid failures. In this review, we provide a comprehensive summary of the advances in reparative therapeutic strategies for MS, including miRNAs and human models. We also discuss their benefits, the likely challenges they face and comment on possible mitigation strategies.

## 1. Introduction

Multiple sclerosis (MS) is a chronic inflammatory and neurodegenerative disease of the central nervous system (CNS) [1], which is estimated to affect 2.8 million people worldwide [2]. It is the most common non-traumatic neurological cause of disability in young people, as it is typically diagnosed between the ages of 20 and 40 [3], which in turn has great impact on the quality of life of patients and their families [4]. The etiology of MS is unknown, but it has been suggested that the disease is the result of a complex interaction between both environmental and genetic factors [5].

MS is highly heterogeneous, and its pathology can vary among patients and even in a single person over time [6]. It is defined as a chronic demyelinating and neurodegenerative disease whose main pathological hallmark is the appearance of confluent demyelinated areas in the white and gray matter of the brain, spinal cord and optic nerve that are indicative of loss of myelin sheaths [7]. Demyelination is accompanied by infiltration of inflammatory cells into the brain parenchyma, formation of gliosis and the destruction of oligodendrocytes (OLs), axons and even neurons [7,8].

Interestingly, remyelination is a natural biological process that can totally or partially repair demyelinated lesions, maintaining axonal integrity and attenuating axonal degeneration. This restorative process involves the interplay of different cell types in the CNS, and it is often disrupted in MS subjects [9]. Although MS is a complex disease with different pathological mechanisms, most therapeutic options focus on modulating the immune response to reduce the formation of new lesions and relapse frequency. Most of these therapies are indicated in the treatment of relapsing–remitting MS (RRMS) and demonstrate little or no efficacy in progressive forms of the disease. Some molecules have also been studied for their neuroprotective properties, reviewed elsewhere (i.e., biotin, antibodies targeting LINGO-1, clemastine, alpha-lipoic acid) [10]. As myelin is necessary for the metabolic support of axons and the transmission of action potentials, research efforts should focus on developing remyelinating therapies that could repair damage, restore function and thereby prevent neurodegeneration [11].

The complex and variable progression of MS underscores the need for early and specific biomarkers to improve diagnosis and therapeutic strategies. microRNAs (miRNAs) are small non-coding RNA molecules that regulate gene expression post-transcriptionally and implicated in MS [12]. Most miRNAs are located inside cells, but extracellular or circulating miRNAs can be detected in biological fluids such as plasma, serum, urine and cerebrospinal fluid (CSF) [13]. Circulating miRNAs are promising biomarkers for different pathological conditions, including MS, as they are remarkably stable and easily accessible [14]. In addition, miRNAs are involved in different pathological mechanisms observed in MS, including neuroinflammation [15], regulation of immune cells [16] and repair processes [9,17], which makes them promising therapeutic candidates.

Studying the human brain is inherently complex due to its inaccessibility, and to date research in the field has depended heavily on model organisms [18]. There has been an increase in our capacity to understand pathophysiological and remyelination processes in MS using mostly in vitro, ex vivo and in vivo models from rodents. However, there are some limitations as demyelinating disorders in animals are remarkably different from human CNS diseases [19]. No single model replicates all aspects of the disease, but various models are valuable for studying specific processes in MS, like inflammation, demyelination and neurodegeneration [20]. Cells of the CNS can also present transcriptional and functional differences among species [21,22]. Given that MS only affects humans [19], the development of human model systems such as those using induced pluripotent stem cells (iPSCs) present a valuable and reproducible alternative that is more closely related to human pathology and MS phenotypes [23,24].

This review summarizes the latest findings on miRNA research in MS regarding their potential role as biomarkers and promising results for their use as a therapeutic option in MS. Moreover, the most recent and advanced human models that can be useful tools to study reparative therapeutic strategies for MS will be described. The latest approaches combining miRNA therapeutics and iPSC-derived models will be depicted, and their advantages and likely challenges discussed, suggesting possible mitigations and explaining future perspectives.

## 2. Cellular Basis of Neurodegeneration in MS

**Figure 1:**
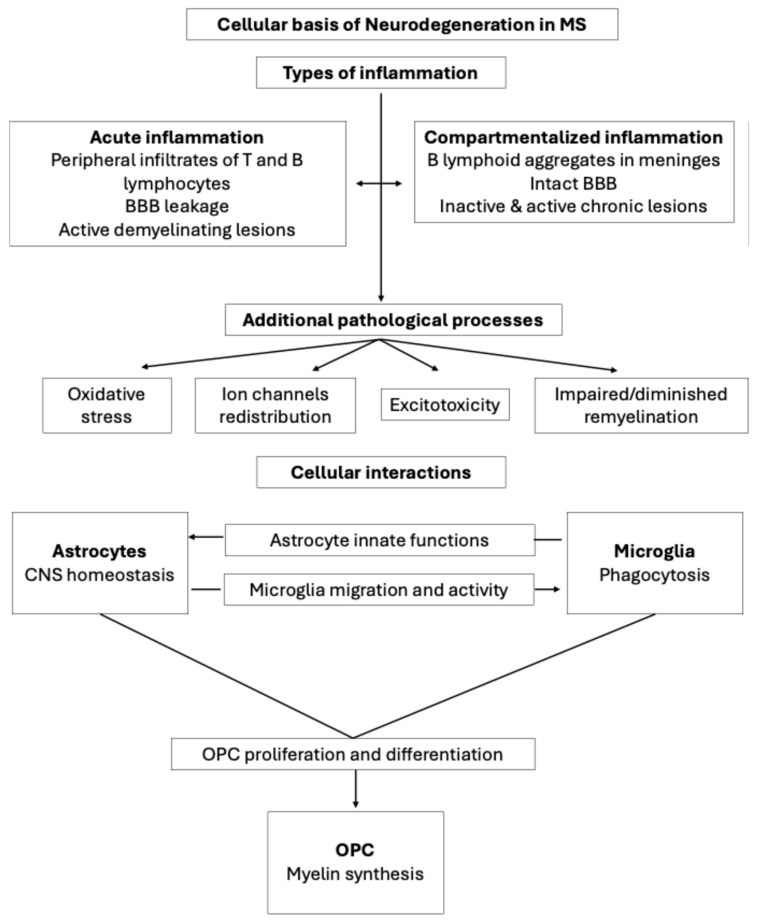
Main pathological events that occur in MS are summarized. The role of astrocytes, microglia and oligodendrocyte precursor cell (OPC) within the central nervous system (CNS) as well as the interactions between them in the context of remyelination are presented. BBB: blood brain barrier.

It is suggested that two types of inflammation might develop in parallel but independently in individuals living with MS (Figure 1) [25]. On one hand, inflammation is mainly observed in acute and RRMS patients. This is characterized by peripheral inflammatory infiltrates of T and B lymphocytes, with intense blood brain barrier (BBB) leakage [6]. This acute inflammation predominantly affects the white matter, forming active demyelinating lesions [25]. On the other hand, although already present in early stages of MS, the second type of inflammation is more pronounced in later stages of the disease and the progressive forms of MS (PMS). This second type is compartmentalized and mainly propagated by B lymphocytes, which appear as lymphoid aggregates in the meninges, adjacent to an intact BBB [26]. This type of inflammation is associated with the presence of inactive and active chronic plaques [25]. The involvement of B lymphocytes in the pathogenesis of MS has acquired importance recently due to the effectiveness of treatments targeting B lymphocytes and neuropathological and serological findings [27].

Other processes can also help explain characteristic pathological findings in MS. Firstly, the chronic inflammatory state in MS is believed to increase the production of both reactive oxygen species and reactive nitrogen species by activated microglia. This toxic environment induces metabolic stress, energy deficiency and loss of neuronal competence, magnifying the existing inflammatory response [28]. Secondly, the chronic inflammation and demyelination has been demonstrated to cause a redistribution of the ion channels, which drives differences in energy demand and consumption and the activation of compensatory mechanisms in mitochondria [29]. Thirdly, extracellular glutamate concentration increases due to neuronal injury and macrophages and microglia activation, causing excitotoxicity. Glutamate excitotoxicity, ion channel redistribution and mitochondria impairment promote an ionic imbalance, which drives axonal damage. This damage occurs during the entire course of the disease and can spread in an anterograde or retrograde manner from the initial site of axonal injury [30].

Although neurons and axons have limited potential for regeneration, ‘shadow plaques’ are evidence that myelin could be repaired during remyelination. Briefly, adult OL progenitor cells (OPCs) close to a plaque are activated in response to the demyelinating insult. In turn they expand and migrate to the damaged area, where they differentiate into mature OLs with the ability to form new myelin sheaths. Notably these new sheaths are often thinner than the ones formed during normal myelination in the development but are functional and supportive of neuronal function [9]. Remyelination typically fails in MS patients, and this natural repair process driven by OPCs is not enough to compensate for the ongoing demyelination in MS [31]. Ageing also affects this process; it is well documented that the maturation of OPCs declines over time, and macrophages also show reduced efficiency in myelin debris clearance [6]. Therefore, new approaches should be exploited to find remyelinating therapies that could restore damage and function, preventing neurodegeneration and alleviating MS symptoms.

### Cell Interactions Within CNS

Both the pathological mechanisms observed in MS as well as the reparative process of remyelination are all consequences of complex interactions among peripheral immune cells and glial cells within the CNS. Recent reviews have delved into the specific roles of different immune cells, OPCs, microglia and astrocytes in the development and pathology of MS [6,26,27,32,33,34]. Here, we will briefly highlight the role of each in the pathology of MS to highlight the complexity of the disease and the importance of using appropriate experimental models (Figure 1).

Glial cells, including OLs, microglia and astrocytes, play critical roles in brain homeostasis by supporting and protecting neurons [35]. Astrocytes, the most abundant cell type of the glia, are highly active in the homeostatic microenvironment of the CNS. Microglia act mainly as phagocytic cells, being responsible for the phagocytosis of degenerated myelin sheaths, while OLs, which are originated from OPCs, produce CNS myelin [36]. All of these cells are extremely plastic and can alter their phenotype to adjust to specific needs within the CNS, and communication amongst them regulates both their activity and function [37]. As these cellular interactions are recognized as vital regulators of myelin status, their implication in a demyelinating disease such as MS is understandable. For instance, OPC proliferation and differentiation into mature OLs is regulated by both microglia and astrocytes [37,38]. Additionally, astrocytes can modulate microglial migration, phagocytic activity and pro- or anti-inflammatory phenotypes, while microglia can regulate the innate functions of astrocytes [37].

Neuroinflammation is a complex process involving the coordinated activity of glial cells. When myelin sheaths are destroyed and accumulated in the extracellular space, microglia are activated and recruited to clean the debris, and primary neuroinflammation advances. Reactive astrocytes are induced by these activated microglia-secreted cytokines [35], and they participate in myelin debris removal [36,38] and the recruitment of more microglia into the lesion [35]. As different stages of neuroinflammation have been described, glial cells modulate their phenotype along this process from amplifying and propagating inflammatory signals to modulate inflammation by intercellular communication and feedback, which confers the pro- and anti-inflammatory aspects of neuroinflammation [39]. It should be mentioned that this inflammatory process is also fueled by the infiltration of peripheral immune cells in the CNS and the increased levels of pro-inflammatory cytokines [40]. The contribution of B and T lymphocytes to the neuroinflammation and pathology of MS has been widely reviewed. Several B cell subsets might act as antigen-presenting cells and pro-inflammatory cytokine-producing cells, while other subsets might present an anti-inflammatory regulatory action [27]. Different types of T cells, such as effector T cells and regulatory T cells, are involved in the development and progression of MS [41]

Remyelination is a complex and multi-cellular driven process [35]. As mentioned, OLs produce the new myelin sheaths wrapping the axons [36]. The proliferation of OPCs and their differentiation into mature myelinating OLs is dependent on astrocytes and microglia. Astrocytes secrete either regenerative or inhibitory factors [35]. On the other hand, microglia promote OPC proliferation and differentiation by means of myelin debris phagocytosis, secretion of regenerative factors and regulation of the extracellular matrix [35]. Availability of lipids is essential for remyelination since myelin has a high lipid content [42] and both astrocytes and microglia also influence lipid metabolism [38].

## 3. microRNAs in MS: Biomarkers and Beyond

Since their discovery in 1993 by Lee et al. [43], miRNAs have been studied in the context of multiple diseases, mainly cancer and inflammatory and neurological diseases. Although the number of studies assessing their role as biomarkers clearly eclipses those investigating their therapeutic potential, their association with diseases has been widely demonstrated [44]. Interestingly in MS, studies have identified dysregulated miRNAs in different types of lesions from MS individuals [45,46,47,48,49,50], which might shed light on their role in the disease and expand our understating of MS. Moreover, some miRNAs are known to be involved in the regulation of cells with relevant roles in MS pathology, such as OPCs [51] or immune cells including T cells [52], and even in repair processes [9], which makes them interesting candidates for therapeutic intervention. Here, we outline their role as biomarkers in MS and describe examples of their therapeutic potential in MS to highlight their likely clinical utility in the disease.

### 3.1. miRNAs as Biomarkers in MS

Since miRNAs show high stability in biological fluids [53], their potential as biomarkers in MS has been widely studied. In Table 1, studies carried out analyzing miRNAs in MS in serum [54,55,56,57,58,59,60,61,62,63,64,65,66,67,68,69,70,71,72,73,74,75,76,77,78,79,80,81,82,83,84,85,86,87,88,89,90,91,92,93,94,95,96,97], plasma [98,99,100,101,102,103,104,105,106,107,108,109,110,111,112,113,114], CSF [59,61,66,73,74,93,102,105,106,115,116,117,118,119,120,121,122,123,124,125,126,127,128,129,130,131,132] and urine samples [111,133] have been included, and their main findings and comparisons have been summarized. As depicted in Table 1, most studies have been performed in serum samples of RRMS subjects. Some miRNAs that have been found deregulated in various studies might be miR-146a, miR-155, miR-126-3p miR-150-5p, miR-223-3p, miR-342-3p, miR-191-5p, miR-320b, miR-145-5p or miR-21-5p.

### 3.2. miRNAs with Functional Therapeutic Evidence of Repair in MS Models

The therapeutic potential of miRNAs for remyelination and neurorepair has been less studied. Some promising results have been described in in vitro and in vivo models routinely used in MS. The major findings of these studies, as well as the therapeutic application and experimental models employed, are summarized in Table 2 [51,130,134,135,136,137,138,139,140,141,142,143,144,145,146]. miR-219, miR-125a-3p and miR-146a are the most studied miRNAs in terms of therapeutic intervention. miR-219 overexpression promoted OL differentiation and showed potential in promoting remyelination [136,141,144,146]. On the contrary, miR-125a-3p overexpression impaired OPC maturation and differentiation [130,135]. Studies regarding miR-146a are contradictory [137,139,142]. On one hand, Martin et al. described a reduced demyelination and increased number of myelinating OLs when miR-146a is absent in mice. On the other hand, Zhang et al. observed an enhanced remyelination when miR-146a mimic was administered. The use of lentivirus, mimics or antagomiRs and the generation of mutant mice are the most common ways of manipulating miRNA expression.

## 4. Experimental Models for Studying Neurorepair in MS

**Figure 2:**
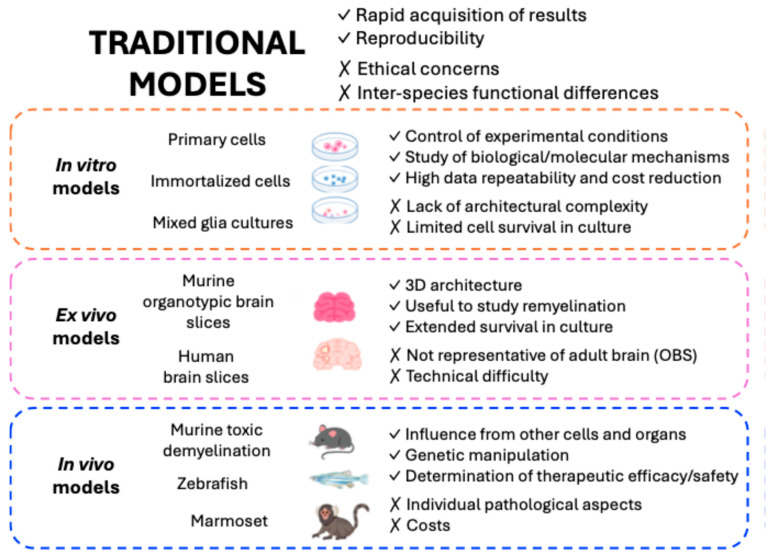
Traditional experimental models for studying neurorepair in MS. General advantages and limitations of traditional models. Different alternative models and their specific advantages and limitations are presented. A tick (✓) indicates advantages; a cross (✗) indicates limitations. OBSs: organotypic brains slices. Figure created using Canva (www.canva.com (accessed on 17 July 2025)).

**Figure 3:**
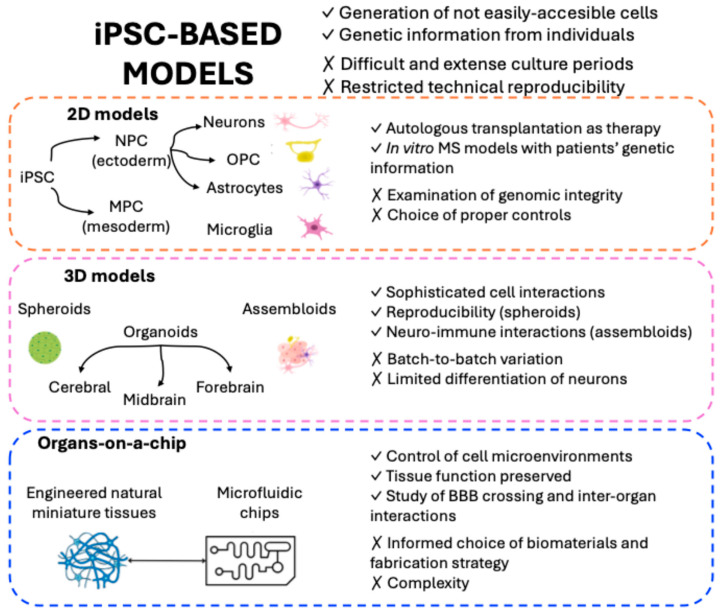
Advanced human models for studying neurorepair in MS. Main advantages and limitations of iPSC-based models. Alternatives models and their specific advantages (✓) and limitations (✗) are presented. iPSC: induced pluripotent stem cells; NPCs: neural progenitor cells; OPCs: oligodendrocyte precursor cells; MPC: mesenchymal progenitor cells; MS: multiple sclerosis; BBB: blood–brain barrier. Figure created using Canva (www.canva.com (accessed on 17 July 2025)).

Study of the processes and coordinated cellular mechanisms that drive pathology in MS can be difficult due to the inaccessibility of the human brain, and much work has focused on the use of in vitro models [147]. Therefore, experimental models that can recapitulate, at least in part, these interactions and crosstalk are necessary and potentially groundbreaking for the appropriate study of neurorepair. Although there is not a single model to cover the wide spectrum of MS pathology, more advanced models have been developed recently [23]. In this section, we review several options for the study of neurorepair, from traditional models including murine in vitro and in vivo models (Figure 2) to advanced human models such as organs-on-a-chip (OoAC) approaches (Figure 3) and their main advantages and limitations in MS research.

### 4.1. Traditional Models

#### 4.1.1. In Vitro and Ex Vivo Models

One of the benefits of using in vitro models is that the experimental conditions can be strictly controlled, assuming that only specific cell types are present. In MS research, primary cells such as neurons, OPCs, astrocytes or microglia can be easily obtained from rodent brains or even human post-mortem tissues and cultured alone to study the biological and molecular mechanisms involved in remyelination and neurorepair [147]. However, since they are primary cells, it is necessary to establish new cultures from time to time to ensure continuity. To circumvent this limitation, some immortalized cell lines have been developed and introduced into the MS field, such as neuronal-like cell lines (HCN, NT2 and SHSY5Y), OPC/OL cell lines (CG4, 6E12 and Oli-Neu) or astrocyte cell lines (C8-D1A and NHA) [23]. Immortalized human microglia cell lines have also been generated, such as HMC3 [148]. However, several studies have reported inconsistent responses after immortalization or challenges in achieving proper differentiation [23].

Although the use of specific cell types can be useful for studying certain molecular mechanisms, in vitro models that combine several CNS cell types have been developed to mimic more precisely the complex interactions within CNS during remyelination. For instance, the murine mixed neuron-glia model is an effective approach to study basic cell responses in the CNS since they include the diversity of CNS cells [149]. Using embryonic mice, cultures of myelinated axons can also be obtained in a consistent and repeatable manner [150]. This technique produces dense axonal cultures featuring thick myelin, abundant synapses and numerous nodes of Ranvier, making it suitable for both morphological and biochemical analyses in neuropathological research [150]. Moreover, these cultures show spinal-like electrical activity and innate CNS immune functions and have been tested for gene-silencing strategies [151].

Bidimensional (2D) monolayer cultures are not able to reproduce the architectural complexity of the CNS; organotypic brain slices (OBSs) from different animal sources, mainly murine, have been widely used to screen promising candidates to promote remyelination. These can represent different regions of the CNS and facilitate experimental manipulations [23]. OBSs can also be kept ex vivo for several months, allowing the study of myelination [152]. Moreover, as demyelination can be induced in OBSs by using toxins such as lysolecithin (LPC) [153] or immune-insults [154], they are a very suitable tool to assess how the brain endogenously remyelinates in a system where cell–cell interactions are preserved. For instance, in the context of remyelination in MS, OBSs have been used to test and demonstrate that fingolimod, one therapeutic option for RRMS [155], enhances remyelination by modulating multiple neuro-glial cell responses [156]. It is worth noting, however, that OBS cultures are usually obtained from early postnatal murine tissue, which might not properly mimic what occurs in an adult brain. Recently, OBSs from adult cerebellar tissue have been optimized, offering a new tool for the study of myelin degeneration and repair [157].

Last but not least, due to discrepancies among species, human brain slices (HBSs) can be used in MS research. Although not widely available, there are feasible protocols to obtain HBSs from post-mortem biopsies or from patients undergoing surgery [147]. In this way, neurons and glia can survive for long periods of time, maintaining their morphological and physiological characteristics [158]. Recently, Plug et al. showed that HBSs allow for testing potential therapeutic options in MS, including gene therapy approaches, as they are susceptible to LPC-induced demyelination ex vivo and were suitable for multi-electrode neuronal recordings. Therefore, HBSs are a highly valuable tool in preclinical research to study mechanisms in living human tissue, including remyelination in MS [159].

#### 4.1.2. In Vivo Models

The use of in vitro models avoids undesired influence from other cells, for example, peripheral immune cells, which do not replicate what occurs in vivo [147]. Although numerous in vivo models of MS have been developed, the complexity and heterogeneity of the disease preclude any single model from reproducing its full pathological spectrum [20]. This highlights the importance of choosing an adequate experimental model to unravel specific MS research questions. Experimental autoimmune encephalomyelitis (EAE), which requires an external immunization to recapitulate both the inflammation, demyelination and neurodegeneration typical in MS, represents the most used model and gold standard in the field [23]. There are other models of MS where demyelination is induced by viral infection with Theiler’s Murine Encephalopathy Virus, murine Hepatitis Virus or Semliki Forest Virus. These viral models and the EAE model essentially replicate demyelination with an autoimmune aspect [23].

But there are models that do not emulate the autoimmune pathological aspect of MS, offering the opportunity of studying the biological mechanisms and metabolic processes involved in myelin regeneration without the complexity of inflammatory processes driven by adaptive immunity [20,160]. Toxic agents such as cuprizone (CPZ), LPC, ethidium bromide (EB) or lipopolysaccharide are mainly used to induce demyelination in these models [23]. One of the most widely used toxic demyelination models is based on the systemic administration of CPZ [161], where exposure to the toxin for 4–6 weeks induces acute demyelination, while prolonged exposure of up to 12 weeks leads to chronic demyelination in both white matter tracts and cortical gray matter [160]. These toxic agents can also be injected into specific areas of the spinal cord, optic nerve, cerebellar or subcortical white matter to generate focal areas of demyelination [160]. This approach offers the advantage of ensuring a coordinated rapid demyelinating event, good reproducibility and a clearly defined anatomical location of the demyelinated area [23]. Lastly, transgenic mice have been generated to study the role of different factors in CNS damage. For instance, mutant mouse models with altered expression of myelin proteins such as myelin basic protein (MBP) or myelin proteolipid protein develop demyelination [19]. There are also models with inducible OL ablation that have been used to investigate de-/remyelination [160].

Another key model in neuroscience research has been the zebrafish as these vertebrates present unique advantages. Zebrafish have genetic, anatomical and physiological similarities to humans, including homology with myelin biology in mammals [162]. Secondly, due to their small size, transparency and quick development, they provide a high-throughput tool for testing potential remyelination therapies. This has been done in the context of looking for enhancers of OPC recruitment and differentiation [163]. Thirdly, their maintenance is more economical than other animal models, and lastly, it is possible to create transgenic zebrafish lines expressing fluorescent markers to trace OPCs for focused studies on remyelination [19]. Of note, as is the case in murine models of MS, some studies have induced demyelination in zebrafish by using CPZ [164], EB [165] or LPC [166].

Last, EAE can also be induced in the common marmoset. This is a validated model for MS that might be used for investigating pathological mechanisms and testing new potential drugs and protocols since it presents focal inflammatory demyelinating lesions [167]. The detection of remyelination in vivo using magnetic resonance imaging (MRI) might be challenging. Recently, this model was used to investigate how sensitive 7T MRI is to characterize remyelination. Donadieu and colleagues compared radiological and pathological findings to determine that MRI had high sensitivity and specificity for the detection of remyelination, which will have implications for future preclinical studies of remyelinating candidates [168].

#### 4.1.3. Advantages and Limitations

In conclusion, these models are crucial to understanding aspects of the pathophysiology of MS. In vitro models offer high data repeatability and the potential to save expenses and experimental time, whereas in vivo models are required to test the efficacy and safety of potential therapies since the response of tissues and the whole organisms as well as behavioral changes can be observed [23]. In addition, the ability to generate transgenic mice has contributed greatly to understanding the contribution of specific molecules and cell types in MS, providing new therapeutic targets and the creation of humanized models by the expression of different human-derived molecules associated with the disease [23].

However, these also present certain limitations, prompting a growing interest in alternative models and techniques to compensate for, or complement, these shortcomings. The etiology of MS differs between humans and animals [147]; functional differences and gene expression patterns of CNS cells exist between species and results cannot always be extrapolated [169]. For example, treatments that have tested positively in in vivo models have failed in clinical trials [20], including atorvastatin, lipoic acid or opiniciumab, among others [170]. In vivo models inherently entail a high economic cost and ethical concerns [147]. Animal welfare should be given the highest priority in research, and use of the 3Rs (reduction, replacement, refinement) should be fulfilled in biomedical research as much as possible [171].

### 4.2. Advanced Human Models

#### 4.2.1. iPSCs and iPSC-Derived Neural Models

Although CNS human primary cells can be obtained from human post-mortem tissues, these cells do not recapitulate the initiation of the disease. In 2006, Takahashi and Yamanaka were able to induce pluripotent stem cells from mouse embryonic or adult fibroblasts by introducing the following factors: Oct3/4, Sox2, c-Myc and Klf4. These cells were designated as iPSCs, expressing embryonic stem cell marker genes and presenting potency and self-renewal [172]. Since their discovery, iPSCs have emerged as an irreplaceable tool for basic research, disease modeling and drug discovery. They offer the opportunity of procuring different types of cells that are not easily accessible, such as the ones within the CNS [147]. Moreover, iPSCs preserve the genetic information from individuals, which can be helpful in linking genetic variants to cellular phenotypes in the context of disease [23].

iPSCs are valuable resources in MS research [173], as in recent years different research protocols have been developed to differentiate iPSCs into neural progenitor cells (NPCs). For example, the combined application of Nogging and SB431542, both SMAD pathway inhibitors, is sufficient to rapidly and completely induce neural conversion [174]. From these NPCs, different types of neurons (midbrain dopaminergic neurons, cortical neurons, spinal motor neurons, sensory neurons) [175], OLs and astrocytes can be obtained. OPCs can be observed within 50 days, and they can be differentiated into myelinating OLs [176]. There are also protocols to obtain and isolate astrocytes, which can be further used for co-cultures or neurotoxicity assays [177]. Microglia, from mesodermal origin, can be differentiated from iPSC-derived myeloid progenitors to generate ramified microglia with motile processes and the ability to phagocytose [178].

Initially, a favorable option was to differentiate iPSCs into simple 2D culture, but the recent development of complex tridimensional (3D) models offers the opportunity to explore more sophisticated cellular interactions [179] and test therapeutic options on models that more accurately replicate in vivo conditions [147]. Among these 3D models, spheroids, organoids and assembloids have demonstrated their potential for studying neurological diseases including MS [23].

Spheroids can be considered the simplest 3D model generated from iPSCs since they are spherical cellular units, usually cultured as free-floating aggregates [180]. Spheroids can be generated by differentiating neural stem cells (NSCs) into different types of cells, such as neurons and glial cells. While they lack structural complexity, their robustness and reproducibility make them a powerful tool for drug screening, having been demonstrated to have functional features including intracellular calcium oscillations [181].

Organoids are cells grown in 3D that form structural units to partially mimic a specific organ, both in structure and function [180]. Cerebral organoids and region-specific brain organoids can be generated. Cerebral organoids are derived from an unguided neural induction procedure, and they comprise independent and distinct tissues reflective of the brain area, showing well-organized apical-basal polarity and neuronal migration [182]. To date, only midbrain and forebrain organoids are the only region-specific brain organoids to have been generated [182]. Different protocols to obtain functional human midbrain organoids have been proposed, in which the introduction of specific signals at defined timepoints drives iPSCs towards a midbrain fate. Some of these propose specific procedures and adaptations to obtain numerous organoids of high quality in the same culture that can be further histologically analyzed [179]. Simultaneous inhibition of SMAD and Wnt signaling pathways might already promote the specification of a dorsal forebrain fate, and the addition of the Shh pathway activation produces ventral forebrain organoids [183]. It is important to choose the most appropriate brain organoid depending on the neurological disease to be studied.

Interestingly for MS and neuroreparative research, Madhavan et al., in 2018, described for the first time the generation of OL and myelin in spheroids derived from human iPSCs [184]. Briefly, the molecular characteristics of maturing OLs and early myelin were found after 20 weeks in culture, and further maturation and myelin compaction was observed by week 30. Moreover, the rate and extent of OL generation and maturation was significantly increased using promyelinating drugs [184]. Since this initial study, different protocols have been optimized to reduce the time for the differentiation of cultures [23].

Assembloids represent a recent advancement in brain organoid technology, as they allow the integration of multiple brain regions and/or distinct cell lineages [23]. This combination allows the functional modeling of processes including cell migration and, importantly in MS, neuro-immune interactions [182]. As mentioned previously, microglia, which play fundamental roles in MS, are of mesodermal origin, so they are not present in brain organoids [178]. Recently, a novel method for the generation of neuroimmune assembloids using cortical organoids and microglia was proposed. Briefly, Kalpana et al. described two different methods to integrate microglia: direct addition of microglia progenitors into the organoids and the aggregation of microglia and dissociated NPCs in a defined ratio [185].

#### 4.2.2. Organs-on-a-Chip and Microfluidic Systems

The combination of advances in stem cell technology, tissue engineering and microfabrication in recent years has led to a next-generation experimental tool to study human pathophysiology and therapeutics, OoAC [186]. These are systems where engineered or natural miniature tissues, such as organoids, are grown inside microfluidic chips to better control cell microenvironments and preserve tissue functions [186]. In this way, the in vivo physiology of an organ might be recapitulated using different cell types and an extracellular matrix in miniaturized devices [187]. They also allow real-time imaging and in vitro analysis of biochemical and metabolic activities of living cells in a tissue and organ specific manner [188]. The type of biomaterial and fabrication strategy employed in the microfluidic system is crucial for the success of OoAC. Certain biomaterials, such as polydimethylsiloxane, are routinely used due to their ease of fabrication but, lately, combinations of different materials have started to emerge [187]. Not only have OoAC shown their utility in personalized medicine and drug screening but they can also be used to model and unravel communication between organs via exosomes [189].

Brain-on-a-chip (BoC) technology has opened new venues in the development of neurological therapies, offering an innovative tool to reproduce the complexity of the human brain. By combining human cells and dynamic systems, it has been possible to generate microphysiological representations of specific brain regions with their function and architecture preserved, including neuronal compartments, fluid conditions and the integration of non-neuronal cells such as glial cells or vasculature [190]. An important challenge in CNS therapeutics is determining the capacity of a therapeutic to cross the BBB and get to the brain, so mimicking the physiology and function of the BBB artificially has been an obstacle in neurosciences. BoC technologies are emerging as microfluidic tools to combine brain and BBB to allow the screening of new brain-directed therapeutic candidates [191]. Moreover, some attempts have been made to represent the whole body-on-a-chip [192], such as the study of Novak et al., where, by combining cutting-edge techniques such as robotics, culture automatization and in situ microscopy imaging, up to ten organs could be cultured on a chip [193].

#### 4.2.3. Human Models with MS Samples

Patient-specific iPSCs can be utilized to create in vitro MS models [147]. Song et al. generated the first iPSC line from an RRMS patient in 2012 [194], and since then more than 50 iPSC lines have been developed [23]. These models represent a valuable tool for drug discovery and cell therapy [147]. In Table 3, most studies of iPSCs generated from MS patients are described, including the starting cell type, reprogramming method and induced cell type [24,194,195,196,197,198,199,200,201,202,203,204,205,206,207,208,209,210,211,212,213,214,215,216,217,218,219]. In summary, most of the donors presented an RRMS phenotype, although lines from PMS have also been generated. The main starting cell types were peripheral blood mononuclear cells and skin biopsies, while some studies have used renal proximal tubule epithelial cells [198,217] or even menstrual blood-derived stromal cells [212]. Sendai virus is the most used method for reprogramming somatic cells, followed by episomal vectors. Some studies have obtained promising results for the study of remyelination and neurorepair. In 2014, Douvaras et al. generated functional OLs from PPMS patients with myelinating capacity [218]. Nishihara and colleagues generated brain microvascular endothelial cells (BMECs) to analyze barrier properties, showing that MS-BMECs presented impaired junctional integrity and an inflammatory phenotype, which could be modulated by the activation of Wnt/β-catenin signaling [204]. Therapeutic interventions have also been tested since propionic acid promoted neurite recovery in induced primary neurons [198]. Finally, MS-organoids containing microglia have recently been implemented [195] and, curiously, the effects of microgravity have been studied by sending them to the International Space Station, where maturation-associated genes were more expressed [196].

#### 4.2.4. Advantages over Traditional Models

In conclusion, iPSCs present numerous advantages over other models. iPSCs maintain the genetic fingerprint of the donor, making them an attractive tool in MS research since it is considered to have a considerable genetic contribution [147]. This could offer the opportunity of implementing disease modeling and drug discovery [173] by helping determine which variant of genes can be involved in the pathology [147]. Additionally, it was recently reported that iPSCs constitutively express the progesterone receptor, which, given the neuroprotective effects of progesterone, enhances the fidelity of iPSC-based models for recapitulating molecular events in disease modeling and therapeutic strategies [220]. Furthermore, the human origin and their ability to be differentiated into almost any cell type provide iPSCs with a therapeutic potential, not only in personalized medicine [173] but also by generating NSCs and OPCs for autologous transplantation [23]. They also offer the opportunity of investigating other aspects of disease, such as interactions between T or B lymphocytes and CNS cells [147], assessing the influence of epigenetic factors or studying the role of mitochondrial failures or the potential of DNA deletions in neurons [194]. Finally, the use of iPSCs helps reduce the number of animals in biomedical research and introduces models that more accurately reflect the disease in a human context.

## 5. Therapeutic Strategies for Neurorepair in MS

### 5.1. miRNAs as Therapeutic Tools

**Figure 4:**
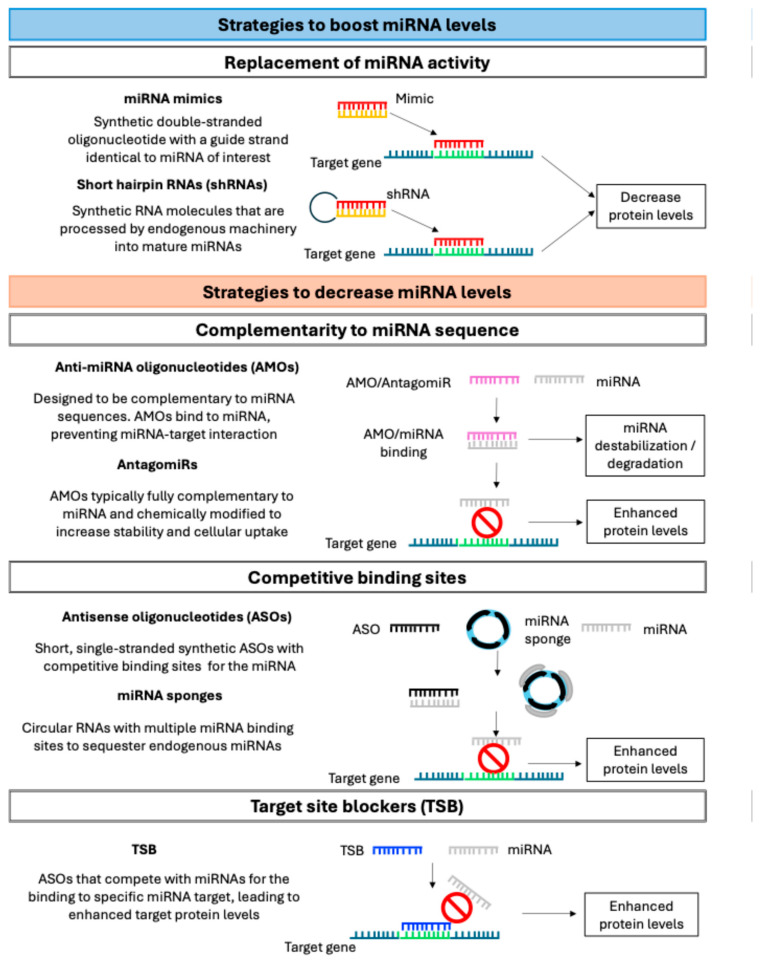
miRNA therapeutics strategies. Main approaches to boost or decrease miRNA levels are presented. miRNA: microRNA; shRNA: short hairpin RNA; AMOs: anti-miRNA oligonucleotides; ASOs: anti-sense oligonucleotides; TSBs: target site blockers.

The aim of miRNA therapeutics is to either boost or decrease the levels of a specific miRNA [221]. By enhancing miRNAs that behave as pathological suppressors or reducing miRNAs that act as pathological drivers, the miRNA expression changes observed during pathological processes might be reversed (Figure 4) [222].

To do so, several RNA-based therapies have been designed [223]. Two approaches can be followed to enhance the level of an miRNA. Firstly, miRNA mimics, which might replace the activity of endogenous miRNAs, are synthetic double-stranded oligonucleotides with a guide strand that is identical to the miRNA of interest [224]. Secondly, short hairpin RNAs (shRNAs) are synthetic RNA molecules that are processed by the processing machinery of the miRNA pathway into mature miRNAs to also replace miRNA expression [221]. In addition, several miRNAs can be co-expressed under the control of a single promoter to modulate a broad set of gene transcripts in one treatment [221].

To repress the function of an miRNA that is overexpressed, different approaches might be implemented. Firstly, anti-miRNA oligonucleotides (AMOs) are synthetic oligonucleotides designed to be complementary to miRNA sequences, hybridizing with them to form a duplex. This binding prevents the miRNA from interacting with its mRNA targets and, in some cases, may lead to destabilization or degradation of the miRNA [53]. AntagomiRs are a specific type of AMO, typically fully complementary to the miRNA and chemically modified to increase stability and cellular uptake [224]. Secondly, the introduction of competitive binding sites for the miRNA might also repress miRNA function. These binding sites can be designed as short, single-stranded synthetic antisense oligonucleotides (ASOs) [221] or as miRNA sponges, such as circular RNAs, which contain multiple miRNA binding sites to sequester endogenous miRNAs [222]. Thirdly, target-site blockers (TSBs) are ASOs that specifically compete with miRNAs for the binding to specific mRNA of an miRNA target, preventing them from binding to those sites and ultimately leading to enhanced target protein levels of that specific target [225]. For example, arginase-2 can be protected from miR-155 action using TSBs, enhancing anti-inflammatory characteristics in macrophages

RNAs present certain characteristics, such as instability, negative charge and hydrophilic nature, that might inhibit or impede their diffusion through the cell membranes [223]. To improve cellular stability or uptake of RNA-based therapies, several strategies have been designed. Locked nucleic acids are chemically modified RNA nucleotides with at least one type of stabilizing element [224] that exhibit specific binding, stability and nuclease resistance [53]. Viral vectors might also be used to constitutively express or inhibit an miRNA by transducing cells with some types of virus, such as adeno-associated virus (AAV), herpes simplex virus or lentivirus (LV) [224]. In addition, therapeutic intracellular miRNA delivery can be enhanced using carriers, including liposomes, polymers, hydrogels, functionalized metals or extracellular vesicles (EVs) [223]. EVs might be the most promising particle-based strategy as they are biological mediators of intracellular signaling, transferring proteins, lipids and genetic material like miRNAs between cells [9]. Some studies have demonstrated communication between CNS cells via microglia-EVs containing miRNAs including miR-23a-5p or miR-146a, promoting white matter repair or the loss of excitatory synapses, respectively [226,227]. Regarding scaffolding systems, a fiber-hydrogel scaffold delivering miR-219/miR-338 mimics was proven to enhance remyelination in a model of spinal cord injury [228]. Consistent with these findings, the incorporation of miR-219/miR-338 into a nanofiber-based device enhanced OPC differentiation [229].

### 5.2. Application of Human Models to Explore Therapies

The potential of iPSC-based models in CNS therapeutics is unquestionable. Stem cell transplantation holds a promising regenerative potential itself since it aims to rescue CNS cell loss and impaired remyelination. Hematopoietic stem cells, mesenchymal stem cells (MSCs) and NSCs have been used in preclinical and/or clinical studies for the treatment of demyelination, although NSCs are expected to have greater effects in PMS (237). As NSC availability is limited, the generation of iPSCs from somatic cells and their further differentiation to NSCs or OPCs presents a new opportunity in stem cell therapy [230]. Notably, neurotrophic support and immunomodulation are also crucial to achieve beneficial effect rather than cell replacement alone [230]. This was observed in a study where murine iPSC-derived NPCs were transplanted intrathecally after disease onset in an EAE model. The secretion of leukemia inhibitory factor by NPCs ameliorated clinical and pathological features, promoting survival, differentiation and the remyelination capacity of OPCs and OLs [231]. Transplantation of iPSC-derived NSCs also reduced T lymphocyte infiltration and alleviated white matter damage in EAE [232]. One example in humans is the STEMS Phase 1 clinical trial, where 12 PMS patients were intrathecally transplanted with human fetal NPCs (hfNPCs). No severe adverse reactions were observed at 2-year follow-up, which supports a favorable short-term safety and tolerability profile but does not establish long-term safety. Interestingly, a lower rate of brain atrophy and increased anti-inflammatory and neuroprotective protein levels were detected in the CSF of those patients who had received the highest hfNPC dose [233].

iPSC-based experimental models offer a new research tool to delve into better demyelination and remyelination strategies in MS, including patient’s genetic information [23]. The acquisition of immunocompetence by the incorporation of functional iPSC-derived microglia is essential to obtain a better representation of MS since all CNS cell types interact in both pathological and reparative processes. Since the development of iPSC technology, some protocols have started to implement immunocompetent brain organoids. For instance, Fagiani et al. described a protocol to accelerate OL differentiation and generate forebrain organoids with microglia. Over 8 weeks, these organoids contained mature CNS cell types with transcriptional profiles similar to the adult human brain. Moreover, their exposure to inflamed CSF from MS patients with MS promoted neurodegenerative phenotypes and intercellular interactions [195]. As previously noted, demyelination is the primary pathological hallmark of MS. Therefore, the induction of oligodendrogenesis and myelination in brain spheroids/organoids is paramount in considering them as tools to mimic MS phenotypes in vitro. In this regard, Madhavan and colleagues proposed for the first time a protocol to generate oligocortical myelinated spheroids and tested promyelinating drugs on them, which highlighted their potential for disease modeling and therapeutic discovery [184]. Since then, other studies have been proposed to reduce the time to obtain myelinating cultures [23]. The implementation of immunocompetent myelinating organoids and the ability of these cultures to produce myelin will provide the basis for the future development of a demyelinating model (i.e., by adding LPC), which will be useful to extrapolate the procedure to other neurological and demyelinating conditions such as spinal cord injury, stroke, traumatic brain injury or Alzheimer’s disease.

iPSC-based models present other features that can be exploited for MS research. iPSCs originating from one patient allowed the generation of both CNS cells and immune cells; therefore, assembloids or OoAC technologies might allow the study of interactions between CNS and T or B lymphocytes, mimicking the in vivo pathological mechanisms more accurately [147]. On the other hand, organoids induced from H9 human embryonic stem cells were cultivated up to six months, observing MBP and OLs, which might indicate that these mature human cerebral organoids could be used as tools to study the development of human brains and to model demyelination caused by brain injury, hypoxia, toxins or various autoimmune disorders [234]. Lastly, it has been suggested that cellular senescence is an active process in PMS that may contribute to impaired remyelination. iPSC-derived NPCs from PPMS patients exhibited cellular senescence markers when compared to control NPCs, and treatment with rapamycin restored the NPC-mediated support for OLs. These results highlighted the appropriateness of iPSCs to mimic pathological findings observed in specific MS phenotypes [235].

### 5.3. Translational Potential in MS

In this section, we associate miRNAs and iPSC-based models to highlight their combined potential for the research of reparative therapies in MS. Epigenetics has been involved in iPSC technology since the very beginning as it affects iPSCs’ reprogramming efficiency and influences their differentiation. The differentiation course could be oriented into a specific lineage based on the epigenetic memory of the source cells, or the acquisition of new cell phenotypes can be promoted due to epigenetic aberrations during reprogramming [236]. Specifically, miRNAs have been suggested to play an important and critical role in OL biology and serve to improve the levels of transcriptional modulators during lineage-specific choices [237]. Interestingly, nuclear reprogramming might allow the generation of genetically matched but epigenetically distinct cells, which could allow the functional study of epigenetic alterations in MS. The potential to reset epigenetic abnormalities has already been demonstrated in cancer research [238]. Moreover, Tiane et al. manipulated cellular differentiation and myelination in vitro in human iPSC-derived OLs by altering the DNA methylation state within the promotor region of MBP, suggesting a potential approach to enhance remyelination [239].

Some examples of promising results regarding miRNA therapeutics in organoid models in other diseases can also be found. One study established a novel robust bioengineering platform to produce RNA agents that were effective in the management of tumor progression, showing the efficacy of miR-1291 in the inhibition of pancreatic cancer in patient-derived organoids [240]. Another study using senescence markers showed that a hydrogel composed of senescence-targeting miR-24 and synovial mesenchymal stromal cell organoids had therapeutic effects on cartilage repair in an osteoarthritic microenvironment, enhancing regenerative capacity by modulating cellular senescence [241].

Regarding remyelination, some studies can be highlighted. miR-219 induced the transformation of murine embryonic stem cells into OL lineage cells and miR-219-OPCs transplantation promoted remyelination, enhancing the proliferation of endogenous NPCs after chronic demyelination [143]. In addition, when used to investigate the effect of 4-aminopyridine on neuronal activity and neurogenesis in cerebral spheroids derived from human iPSCs, a significant decrease in miR-135a levels was observed, which had been previously observed in exercise-induced neurogenesis [242]. This might indicate a potential correlation between in vivo and in vitro findings, which might support the use of these models incorporating miRNA targeting in MS research. Furthermore, cerebral organoids can be used to study EV-associated miRNAs involved in neurodegenerative diseases and neurotoxicity. EVs released by human cerebral organoids contained sufficient levels of miRNAs to be confidently quantified, and those miRNAs were related to neurodegenerative disease and nervous system signaling according to pathway analyses [243]. Exosomes from human umbilical cord MSCs stimulated myelin gene expression in OPCs, activating signaling pathways via miR-23a-3p. Interestingly, in the EAE model, treatment with these exosomes enhanced neurological function and facilitated remyelination [244]. Lastly, miRNA candidates have been proposed as potential safety biomarkers of chemically induced neurodegeneration after being detected following secretion by human iPSC-derived neurons [245].

## 6. Challenges and Future Perspectives

Once the potential of miRNA study in iPSC-based models has been explained in the context of remyelination and neurorepair, this section will briefly summarize the major limitations of the application of miRNA therapeutics and the use of advanced human models in research as well as potential mitigations and future perspectives in the field.

### 6.1. Limitations and Challenges in miRNA Therapeutic Application

miRNAs can target hundreds of different genes; thus, they can cause undesirable off-target effects, as the manipulation of the activity of a single miRNA can result in the dysregulation of several genes with unrelated functions to the desired therapeutic effect [9]. This of course can lead to toxicity and reduced therapeutic efficacy [246]. The dosage of miRNA therapeutics can also lead to unpredictable off-target effects and may impact genes that are not directly targeted. Furthermore, therapeutic miRNAs can compete with endogenous miRNAs for cellular resources, triggering potentially toxic off-target effects on multiple signaling pathways [222]. To mitigate this off-target effect, the design of mimics and inhibitors with improved specificity can be pursued [53], and appropriate dosing can be achieved by combining cooperating miRNAs to keep the doses of individual miRNAs as low as possible [222].

Treatment strategies involving miRNAs should consider the appropriate disease stage and cell-specific targeting to maximize therapeutic potential. miRNA expression might have tissue specificity, with altered expression levels across different developmental stages, which indicates a dynamic regulation [247]. Therefore, achieving CNS-specific targeting is crucial for miRNA therapeutics to improve efficacy and reduce adverse effects on healthy tissues [53]. The use of vector-based expression systems might assure tissue-specific expression by using promoter sequences [222]. Different studies have analyzed the use of several AAV serotypes and cell-specific promoters to enhance glial cell tropism [248]. Using an LV system, it has been possible to specifically target astrocytes and, moreover, to target brain circuits affected in CNS diseases, achieving a broad distribution [249]. Another approach has incorporated an on-switch to allow temporal control in astrocytes [250]. Viral transduction efficiency in microglia is relatively low. Recently, some advancements have been made to enhance this process by the co-delivery of Vpx, a protein that facilitates LV reverse transcription, packaged in virus-like particles with LV into iPSC-derived microglia [251].

Another common challenge in drug discovery in CNS diseases is the mode of delivery as the therapeutic agent should cross the BBB. This acquires even more importance in PMS as the BBB is less porous than in RRMS [9]. Many molecules encounter difficulties in reaching the CNS through the BBB due to their rapid removal from endothelial cells by transmembrane proteins. These obstacles might be overcome by using carriers or chemically modifying miRNAs [252]. For example, EVs overexpressing miR-219 were more effective at crossing an in vitro model of BBB than liposomes and nanoparticles, and when administered intranasally in the EAE model they induced increased OPC differentiation and improved clinical scores [253].

One major challenge in miRNA research, not only in the therapeutics area, is the urgent need to minimize variability and optimize normalization methods by the implementation of standardized protocols and the application of quality controls. This is crucial to ensure accuracy and enhance the predictive power of miRNA-based technologies [53]. In the same way, miRNA stability during storage and delivery should be considered, and developing strategies to protect miRNAs from degradation and preserve their biological function will be useful [53].

While the future therapeutic use of miRNAs is promising, few miRNA-based drugs have progressed to a clinical test phase since there are still great practical obstacles to mitigate [222].

### 6.2. Technical and Ethical Obstacles to the Use of Advanced Human Models

The quality of the starting iPSC population is critical for the success of these models, making it essential to adhere to best practices in iPSC culture [173]. In this context, the International Society for Stem Cell Research has proposed a set of recommendations for scientists in basic research laboratories to enhance the reproducibility of stem cell research [254]. Despite these guidelines, there are several technical challenges associated with iPSCs.

Although genetic variability is considered to be an asset, it could also hinder the standardization and reproducibility of outcomes across different cell cultures derived from different donors [147]. Additionally, variability in the differentiation and maturation periods of iPSCs, particularly for neurons, can differ between cell lines. The solution is through the recognition of each cell line’s characteristics and applying isogenic controls [147]. In addition, any study comparing iPSC-based models using multiple iPSC lines should carefully choose the appropriate controls, including normal cell lines generated from close relatives or non-related individuals [194].

The complexity of organoid models hinders their use as drug screening platforms since it is common to observe batch-to-batch variation in size and cell composition, limited differentiation of neuronal cell types and long differentiation and maturation [181]. To mitigate this, the automation of organoid culture and screening processes might be a feasible option to overcome variability, reproducibility and scalability in organoid research [255].

Due to long culture periods, another important aspect to consider when culturing iPSCs is the preservation of their stem cell identity. Sometimes, spontaneous differentiation is observed in these cultures, and morphological assessment of colonies and immunostaining of pluripotency markers should be routinely performed [173]. In addition, iPSC-derived models can display immature functional characteristics and therefore can fail to represent delayed stages of diseases [256]. This aspect is important in MS research as it is a chronic disease usually diagnosed in adult life [194]. The preservation of changes associated with ageing in these models might be achieved by the direct reprogramming of cells, skipping the iPSC phase [147].

Another feature that should be continuously examined is the DNA sequence of each cell line generated as they undergo genetic modifications [23], and some reprogramming vectors might remain integrated in the cell line [194]. This might be overcome by using viral free reprogramming vectors [194].

Not only can technical challenges be associated with these cultures, but also ethical ones. The application of cell and molecular biology methods to modify biological processes and generate organoids makes these entities biotechnology products and, therefore, patentable interventions, which might generate unequal distribution of effective therapies based on socioeconomic status. Organoids do not correspond to any legally regulated category such as cells, gametes, tissues or organs, so defining their legal status is still necessary [257]. Although the purpose of donating cells for organoid generation can still be unclear, the informed consent of individuals to cell donation and the further uses of the generated organoids is always mandatory to protect individuals’ rights to autonomy and self-determination [257]. In addition, gene editing of iPSCs can raise ethical issues since their possible transplantation into humans might eventually lead to other applications that do not protect human dignity [257]. Lastly, the creation of human–animal chimeras by the transplantation of iPSCs or organoids in animal models raises the question of whether or not crossing species is ethically acceptable [257].

### 6.3. Future Perspectives

Despite significant progress, it is important to recognize that we are still in the early stages of harnessing the full therapeutic potential of miRNAs [222]. No miRNA-based therapies have been approved, but several potential candidates have reached phase I and phase II clinical trials. In addition, there are some biotech companies, such as Miragen, Synlogic and Regulus Therapeutics, working on miRNA-based therapies [221].

The pathological mechanisms involved in MS are diverse, but primary demyelination is the main indicator of this chronic disease [8], and an unpaired remyelination is frequently observed in these patients [9]. MS presents clinical heterogeneity [6], and complex CNS interactions are required to orchestrate all these processes [9]. In this complicated context, the emergence of iPSC technology raises new opportunities to explore neurodegeneration and neurorepair and to model the different phenotypes observed in MS. In addition, the implementation of immune-competent myelinating organoids is essential to design a model that might be demyelinated to explore potential reparative therapies. Further developments in assembloids and OoAC will allow the study of inter-organ interactions with remarked relevance in MS, such as the brain-peripheral immune system.

Given the relevance of both miRNA therapeutics and iPSC technology, the synergistic advancements in both disciplines will expand our knowledge of MS, offering new potential options to boost neurorepair and endogenous remyelination. Within this review, several examples of successful enhancement of remyelination have been explained, highlighting the potential of EV-associated miRNAs released by organoids and exosomes released by MSCs to be used in neurodegenerative diseases [243,244]. Moreover, various strategies exist to generate exosomes enriched with pro-remyelination miRNAs [9], presenting an opportunity to further exploit this approach. miR-219/miR-338 have proven their potential to enhance remyelination in different studies by promoting OL differentiation [136,141,144,146]. The emergence of -omics such as single-cell RNA sequencing could reveal new candidates and their cell-specific dysregulation. All the advancements in CNS transduction by using specific AAV serotypes, promoters and temporal and spatial switches should be optimized and exploited in organoids to study new miRNA/target therapeutic candidates, avoiding off-target effects and increasing efficacy sequences [222,248,249,250,251].

## 7. Conclusions

miRNAs are emerging as a promising tool for myelin repair, although their therapeutic application is limited by off-target effects and difficulties in delivery to the CNS. iPSCs offer great potential for studying neurodegenerative diseases such as MS, allowing for the modeling of remyelination and neurorepair processes, including patients’ genetic fingerprints. However, they face technical challenges such as genetic variability and restricted reproducibility. Advances in improving treatment specificity in miRNAs by using specific promoters or even switch-methods to control the expression and the use of organoids or assembloids to model more complex CNS interactions may advance new strategies for remyelination. Therefore, the future of reparative therapies depends on overcoming technical and ethical obstacles, as well as optimizing approaches to maximize treatment efficacy and safety, so more translational and collaborative research is imperative.

## Figures and Tables

**Table 1:** Circulating miRNAs studies in MS.

Study	Year	Major Findings	Reference
**Serum**			
Yañez-Esparza et al.	2025	RRMS vs. HC: miR-143-5p (↑)/miR-145-5p (↓)	[54]
Maimaitijiang et al.	2025	SPMS vs. HC: miR-133b (↓)	[55]
Agostini et al.	2024	SPMS vs. RRMS: miR-34a-5p, miR-103a-3p and miR-376a-3p (↑). cSPMS vs. RRMS: miR-34a-5p, miR-103a-3p and miR-376a-3p (↑)	[56]
Domínguez-Mozo et al.	2024	miR-126-3p: correlation with EDSS at cognitive status at baseline. miR-126-3p, miR-9: correlation with cognitive deterioration at 1 year. miR-9: correlation with sNFL. miR-146a-5p: association with MS phenotype.	[57]
Al-Dahimavi et al.	2024	MS vs. HC: miR-26a, miR-34a, miR-146a (↑)	[58]
Tan et al.	2024	SPMS vs. RRMS: miR-451a, miR-16-2-3p, miR-9-5p, miR-15a-5p, miR-144-3p, miR-100-5p, miR-210-3p (↑)	[59]
Ahmed et al.	2024	MS vs. HC: miR-135-5p (↑)	[60]
Mohammadinasr et al.	2023	RRMS vs. HC: let-7 g-5p, miR-18a-5p, miR-145-5p, miR-374a-5p, miR-150-5p, miR-342-3p (↑)/miR-132-5p, miR-320a-5p (↓)	[61]
Gonzalez-Martinez et al.	2023	NEDA-3 vs. EDA-3: miR-548a-3p (↑)	[62]
Gonzalez-Martinez et al.	2023	Benign MS vs. Not benign MS: miR-320b (↑)/miR-25-3p (↓)	[63]
Geiger et al.	2023	miR-92a-3p, miR-486-5p: associated with greater total white matter lesion volumes. miR-142-5p: associated with total creatinine concentration. miR-92a-3p, miR-142-5p, miR-486-5p: associated with functional connectivity strengths	[64]
Casanova et al.	2023	miR-9-5p: associated with EDSS progression at 2 years. Lower levels of miR-138-5p in NEDA-3 at 2 years. Higher levels of miR-146a-5p and miR-126-3p in CDP progression at 2 years	[65]
Muñoz-San Martín et al.	2022	PPMS vs. OND&RRMS: miR-20a-5p (↑)/miR-320b (↓). PPMS vs. RRMS: miR-26a-5p, miR-485-3p (↓). RRMS vs. OND: miR-142-5p (↑)	[66]
Mancuso et al.	2022	miR-126-3p greatly increased in PML	[67]
Vistbakka et al.	2022	RRMS vs. HC: miR-191-5p (↑). PPMS vs. HCS: miR-128-3p (↑). Temporal changes: miR-191-5p (EDSS, MRI activity) and miR-223-3p (relapse)	[68]
Domínguez-Mozo et al.	2022	miR-146a-5p, miR-9-5p: association with EDSS. miR-146a-5p, miR-126-3p: association with SDMT	[69]
Saridas et al.	2022	miR-146a and miR-155 were significant in the RRMS-Control group for the area under the curve	[70]
Cuomo-Haymour et al.	2022	RRMS&CIS&VI vs. Control: miR-21-5p (↑)/miR-6735-3p, miR-6833-5p, miR-510-3p (↓).	[71]
Khedr et al.	2022	MS vs. HCS: miR-22. SPMS vs. RRMS: miR-22 (↑). miRNA-22: associated with EDSS.	[72]
Perdaens et al.	2020	Rel-MS and Rem-MS vs. HC: miR-15a-3p, miR-24-3p, miR-126-3p, miR-146a-5p, miR-181c-5p (↓). Rel-MS vs. HC: miR-214-3p (↓)	[73]
Domínguez-Mozo et al.	2020	Detection of HHV-6A/B miRNAs. Correlation of: hhv6b-miR-Ro6-2 and hhv6b-miR-Ro6−3-5p, hhv6b-miR-Ro6−2 and miR-U86, hhv6b-miR-Ro6−3-5p and miR-U86	[74]
Zanoni et al.	2020	PMS vs. RRMS: miR-128-3p (↑). No relapses vs. relapses: miR-128-3p (↑)	[75]
Ibrahim et al.	2020	MS vs. HC: miR-300, miR-450b-5p (↓). SPMS vs. RRMS: miR-300, miR-450b-5p (↓).	[76]
Sharaf-Eldin et al.	2020	miR-23a: differentiation of MS from NMOSD and NPSLE	[77]
Shademan et al.	2020	MS vs. HC: miR-146a, miR-155 (↑)	[78]
Senousy et al.	2020	MS vs. HC: miR-137 (↓)	[79]
Ebrahimkhani et al.	2020	miR-150-5p, miR-548e-3p decreased with treatment. miR-130b-3p, miR-654-5p, miR-487b-5p increased after treatment	[80]
Hemond et al.	2019	miR-22-3p, miR-361-5p, miR-345-5p: most valid differentiators of MRI phenotypes	[81]
Vistbakka et al.	2018	RRMS and PPMS vs. HC: miR-24-3p, miR-191-5p (↑). PPMS vs. HC: miR-128-3p (↑)	[82]
Regev et al.	2018	RRMS vs. HC: miR-484 (↑). SPMS vs. HC: miR-320a, miR-320b, miR-320c, miR-484 (↑)/miR-140-5p, miR-142-5p (↓)	[83]
Manna et al.	2018	IFN-MS vs. tn-MS: miR-22-3p, miR-660-5p/let-7b-5p, miR-15b-3p, miR-19-3p, miR-23a-3p, miR-26a-5p, miR-122-5p, miR-142-3p, miR-146a-5p, miR-215-5p, miR-223-3p, miR-320b, miR-320d, miR-451a, miR-486-5p (↓)	[84]
Wang et al.	2017	Rel-MS vs. HC: ebv-miR-BHRF1-2-5p, ebv-miR-BHRF1-3 (↑)	[85]
Regev et al.	2017	miR-142-5p, miR-143-3p, miR-181c-3p, miR-181c-5p: protective correlations with T1:T2. miR-486-5p, miR-92a-3p: pathogenic correlations with T1:T2. miR-375, miR-629-5p: pathogenic correlation with brain atrophy	[86]
Niwald et al.	2017	RRMS vs. HC: miR-326 (↑)/miR-155, miR-301a (↓)	[87]
Sharaf-Eldin et al.	2017	MS vs. HC: miR-145, miR-223 (↑)	[88]
Ebrahimkhani et al.	2017	RRMS vs. HC: miR-15b-5p, miR-30b-5p, miR-342-3p, miR-451a (↑). PMS vs. HC: miR-370-3p, miR-409-3p, miR-432-5p (↑). RRMS vs. PMS: miR-15b-5p, miR-23a-3p, miR-30b-5p, miR-223-3p, miR-342-3p, miR-374a-5p, miR-485-3p (↑)/miR-432-5p, miR-433-3p (↓)	[89]
Selmaj et al.	2017	Rel-MS vs. HC: miR-122-5p, miR-196b-5p, miR-301a-3p, miR-532-5p (↓). Rem-MS vs. HC: miR-122-5p, miR-196b-5p, miR-532-5p (↓). Rem-MS vs. HC: miR-122-5p (↓).	[90]
Vistbakka et al.	2017	PMS vs. HC: miR-26a-5p, miR-191-5p (↑). SPMS vs. HC: miR-26a-5p, miR-191-5p (↑)	[91]
Regev et al.	2016	MS vs. HC: miR-25-3p, miR-140-3p, miR-320b, miR-486-5p (↑)/let-7c-5p, miR-365a-3p (↓). RRMS vs. PMS: miR-27a-3p (↑). RRMS vs. SPMS: miR-27a-3p, miR-376b-3p (↑)	[92]
Ahlbrecht et al.	2016	CIS-MS vs. CIS-CIS: miR-922 (↑)	[93]
Mancuso et al.	2015	MS vs. HC: miR-572 (↓). PPMS vs. HC: miR-572 (↓). Rem-MS vs. HC: miR-572 (↓). SPMS vs. PPMS: miR-572 (↑). Rel-MS vs. Rem-MS: miR-572 (↑)	[94]
Zhang et al.	2014	MS vs. HC: miR-124, miR-146a, miR-210, miR-155, miR-326 (↑). Rel-MS vs. Rem-MS: miR-155 (↑)	[95]
Ridolfi et al.	2013	RRMS vs. HC: miR-15b, miR-23a, miR-223 (↓); PPMS vs. HC: miR-15b (↓)	[96]
Fenoglio et al.	2013	MS vs. HC: miR-15b, miR-223 (↓). PPMS vs. HC: miR-15b, miR-223 (↓)	[97]
**Plasma**			
Elsayed et al.	2024	MS vs. HC: no differences in miR-155. miR-155: positive correlation with TNF-α, INF-ɣ and iNOS, inverse correlation with IL-10, TGF-β and SMAD2	[98]
Al-Temaimi et al.	2024	RRMS vs. HC: miR-23a-3p (↑)/miR-326 (↓); SPMS vs. RRMS: miR-150-5p, miR-320a-3p (↓)	[99]
Al-Temaimi et al.	2024	MS vs. HC: miR-24-3p (↓). RRMS vs. hC: miR-484 (↑). SPMS vs. RRMS: miR-146-5p, miR-484 (↓)	[100]
Scaroni et al.	2022	CI vs. CP: miR-150-5p (↑)/let-7b-5p (↓)	[101]
Zheleznyakova et al.	2021	RRMS vs. N + INDC: miR-215-5p (↓)	[102]
Balkan et al.	2021	MS vs. HC: miR-20 (↑)/miR-26, miR-155 (↓)	[103]
Giuliani et al.	2021	RRMS vs. HC: miR-34a, miR-125a-5p (↑)/miR-146a-5p (↓). After treatment: reduced miR-125a-5p, miR-146a-5p, miR-155	[104]
Muñoz-San Martín et al.	2020	RIS-Conversion vs. RIS-RIS: miR-483-3p (↑)/miR-142-3p, miR-338-3p, miR-363-3p, miR-374b-5p, miR-424-5p (↓)	[105]
Muñoz-San Martín et al.	2019	Gd+ RRMS vs. Gd− RRMS: no differences	[106]
Kimura et al.	2018	MS vs. HC: let-7c, miR-19b, miR-25, miR-92a (↑)	[107]
Saénz-Cuesta et al.	2018	Change of miRNA cargo after fingolimod treatment	[108]
Basnyat et al.	2017	NTZ-MS vs. IFN-MS: jcv-miR-J1-5p (↓)	[109]
Kacperska et al.	2015	Rem-MS vs. HC: let-7a, miR-648a (↓)	[110]
Giovanelli et al.	2015	No differences in Polyomavirus JC miRNA	[111]
Gandhi et al.	2013	RRMS vs. HC: miR-22, miR-30e, miR-140-3p, miR-210, miR-500, miR-547-3p (↑). SPMS vs. HC: let-7a (↓). RRMS vs. SPMS: miR-92a-3p*, miR-135a, miR-454, miR-500, miR-574-3p (↑)	[112]
Søndergaard et al.	2013	RRMS vs. HC: miR-145 (↑)/miR-660, miR-939 (↓)	[113]
Siegel et al.	2012	MS vs. HC: miR-22, miR-422a, miR-572, miR-614, miR-648, miR-1826 (↑)/miR-1979 (↓)	[114]
**CSF**			
Pavlovic et al.	2025	pwMS vs. HC: miR-16-5p, miR-21-5p, miR-150-5p, miR-146a-5p, miR-142-5p, miR-148a-3p, miR-222-3p, miR-92a-3p, miR-342-3p, miR-100-5p (↑)/miR-143-3p, miR-27b-3p (↓)	[115]
Mohammadinasr et al.	2024	RRMS vs. HCS: ebv-miR-BART9-3p, ebv-miR-BART15, hsa-miR-21-5p, hsa-miR-146a-5p (↑)	[116]
Tan et al.	2024	No differences	[59]
Dolcetti et al.	2024	miR-142-3p: correlation with MSSS, disease progression, IL-1β.	[117]
Shademan et al.	2023	MS vs. controls: miR-21, miR-155, miR-182 (↑)	[118]
Mohammadinasr et al.	2023	RRMS vs. HC: let-7 g-5p, miR-18a-5p, miR-145-5p, miR-374a-5p, miR-150-5p, miR-342-3p (↑)/miR-132-5p, miR-320a-5p (↓)	[61]
Zanghì et al.	2023	RRMS vs. OND: miR-106a-5p (↑)	[119]
De Vito et al.	2023	High levels of miR-142-3p	[120]
Muñoz-San Martín et al.	2022	PPMS vs. OND: let-7b-5p, miR-143-3p (↑)	[66]
De Vito et al.	2022	miR-142-3p: correlation with clinical progression. Better response to DMF in ‘low-miR-142’ patients	[121]
Zheleznyakova et al.	2021	RRMS vs. N + INDC: miR-146a-5p, miR-148a-3p, miR-150-5p, miR-181a-5p, miR-29a/b-3p, miR-342-3p (↑)/miR-204-5p, miR-371a-3p (↓)	[102]
Mandolesi et al.	2021	PMS vs. RRMS: let-7b-5p (↓)	[122]
Perdaens et al.	2020	MS vs. SC: miR-150-5p, miR-155-5p (↑)/miR-15a-3p, miR-34c-5p, miR-297 (↓). Rel-MS vs. Rem-MS or SC: miR-24-3p, miR-27a-3p, miR-27b-3p, miR-29c-3p, miR-125b-5p, miR-145-5p, miR-21-5p, miR-146a-5p (↑). Rel-MS vs. SC: miR-124-5p (↓). Rem-MS vs. Rel-MS and/or SC: miR-149-3p (↑)/miR-20a-5p, miR-33a-3p, miR-214-3p (↓)	[73]
Muñoz-San Martín et al.	2020	RIS-Conversion vs. RIS-RIS: miR-144-3p, miR-448, miR-653-3p (↑)	[105]
Domínguez-Mozo et al.	2020	Detection of HHV-6A/B miRNAs. Correlation of hhv6b-miR-Ro6-2 and hhv6b-miR-Ro6−3-5p	[74]
Li et al.	2020	Relapse vs. Remmision and Control: miR-1-3p (↑)	[123]
Kramer et al.	2019	MS vs. OND: miR-181c, miR-633 (↑). SPMS vs. PPMS: miR-181c, miR-633 (↑); SPMS vs. RRMS: miR-181c (↑)	[124]
Muñoz-San Martín et al.	2019	Gd+ RRMS vs. Gd− RRMS: miR-21, miR-146a, miR-146b (↑)	[106]
Bruinsma et al.	2017	MS vs. Controls: Absence of miR-219	[125]
Liu et al.	2017	Rel-MS vs. Rem-MS and HC: miR-590 (↑)	[126]
Wu et al.	2017	Rel-MS vs. Rem-MS and HC: miR-448 (↑)	[127]
Mandolesi et al.	2017	Gd+ RRMS vs. Gd− RRMS and OND: miR-142-3p (↑)	[128]
Quintana et al.	2017	MS vs. OND: miR-30a-5p, miR-150, miR-328, miR-645 (↑)/miR-21, miR-106a, miR-146a, miR-191, miR-199a-3p, miR-365 (↓). LS_OCMB+ RRMS vs. OND: miR-30a-5p, miR-150, miR-645 (↑)/miR-191 (↓)	[129]
Lecca et al.	2016	Active MS vs. Inactive MS and OND: miR-125a-3p (↑)	[130]
Bergman et al.	2016	RRMS vs. NINDC and INDC: miR-150 (↑). CIS vs. NINDC: miR-150 (↑). CIS-CIS vs. CIS-MS: miR-150 (↑)	[131]
Ahlbrecht et al.	2016	CIS-MS vs. CIS-CIS: miR-181c, miR-922 (↑)	[93]
Haghikia et al.	2012	MS vs. OND: miR-181c, miR-633 (↑)/miR-922 (↓). RRMS vs. SPMS: miR-181c, miR-633 (↑)	[132]
**Urine**			
Agostini et al.	2021	miR-J1-5p could be a biomarker to monitor JCPyV infection	[133]
Giovannelli et al.	2015	No differences in Polyomavirus JC miRNAs	[111]

Table 1. Summary of studies analyzing circulating miRNAs in MS using serum, plasma, CSF and urine samples. The main author, year of publication, corresponding reference and the major findings of each study are reported. The table specifies the comparisons performed, where an miRNA (or list of miRNAs) followed by (↑) denotes upregulation and an miRNA (or list of miRNAs) followed by (↓) denotes downregulation for each specific comparison. Associations or qualitative determinations (presence/absence) are also indicated. MS: multiple sclerosis; RRMS: relapsing–remitting MS; HC: healthy controls; SPMS: secondary progressive MS; cSPMS: converted SPMS; EDSS: Expanded Disability Status Scale; sNFL: serum neurofilament; NEDA-3: no evidence of disease activity-3; EDA-3: evidence of disease activity-3; CDP: confirmed disability progression; PPMS: primary progressive MS; OND: other neurological disease; PML: progressive multifocal leukoencephalopathy; MRI: magnetic resonance imaging; SDMT: symbol digit modalities test; CIS: clinically isolated syndrome; VI: viral infection; Rel-MS: relapsing MS; Rem-MS: remitting MS; HHV: human herpes virus; PMS: progressive MS; NMOSD: neuromyelitis optica spectrum disorders; NPSLE: neuropsychiatric systemic lupus erythematosus; IFN-MS: interferon-treated MS; tn-MS: treatment-naive MS; N + INDC: noninflammatory and inflammatory neurological disease controls; RIS: radiologically isolated syndrome; RIS-Conversion: patients who converted to CIS or MS after 5 years of monitoring; RIS-RIS: patients who remained as RIS after 5 years of monitoring; Gd+ RRMS: RRMS individuals with gadolinium-enhancing lesions; Gd− RRMS: RRMS individuals without gadolinium-enhancing lesions; NTZ-MS: natalizumab-treated MS; JC: John Cunningham; MSSS: multiple sclerosis severity score; DMF: dimethylfumarate; SC: symptomatic controls; LS_OCMB- RRMS: RRMS individuals with negative lipid-specific oligoclonal IgM bands; LS_OCMB+ RRMS: RRMS individuals with positive lipid-specific oligoclonal IgM bands; NINDC: non-inflammatory neurologic disease controls.

**Table 2:** Studies of miRNAs with functional therapeutic evidence of repair.

Study	Year	miRNA	Therapeutic Application	Major Findings	Reference
Kornfeld et al.	2024	miR-145	miR-145^+/+^ and miR-145^−/−^ mice	(1) Loss of miR-145 increased remyelination and functional recovery after chronic demyelination with altered presence of astrocytes and microglia; (2) overexpression of miR-145 stunted OL differentiation and survival	[134]
Marangon et al.	2020	miR-125a-3p	Lentiviral over-expression in vivo and ex vivo after LPC-induced demyelination	(1) miR125a-3p over-expression impaired OPC maturation; (2) miR-125a-3p downregulation accelerated remyelination; (3) direct interaction of miR-125a-3p with Slc8a3 and Gas7	[135]
Tripathi et al.	2019	miR-27a	Primary murine OPCs transfected with mimic and inhibitor, primary human OPCs transfected with mimic, intranasal administration of mimic in EAE and CPZ model	(1) Increased levels of miR-27a inhibited OPC proliferation and impaired differentiation of OPCs and myelination; (2) in vivo administration of miR-27a suppressed myelinogenic signals	[51]
Nguyen et al.	2019	miR-219/miR-338	miR-219/miR-338 in microglia and astrocytes from P1–2 neonatal rat cortices	(1) miR-219/miR-338: (a) diminished microglial expression of pro-inflammatory cytokines, (b) suppressed astrocyte activation, (c) enhanced OPC differentiation and maturation	[136]
Zhang et al.	2019	miR-146a	Treatment of EAE mice with miR-146a mimics at day 14 post immunization once a week for 6 consecutive weeks	(1) miR-146a mimic: (a) improved neurological functional outcome, (b) increased the number of newly generated OLs, (c) increased cell number, cytokine level and protein levels of M2 microglia/macrophages, (d) decreased cytokine and protein levels of M1, (e) increased OPC differentiation and remyelination by the reduction of TLR2/IRAK1 signaling pathway activity	[137]
Nazari et al.	2018	miR-219	miR-219-GFP-expressing lentivirus in hiPSCs	(1) Increased expression of pre-OL markers	[138]
Martin et al.	2018	miR-146a	miR-146a^−/−^ mice and CPZ-induced demyelination model	(1) Absence of miR-146a reduced inflammatory responses, demyelination, axonal loss and the number of infiltrating macrophages and increased the number of myelinating OLs	[139]
Ghasemi-Kasman et al.	2018	miR-302/367 cluster	Administration of lentivirus into the corpus callosum of CPZ-induced demyelination mice	(1) Expression of the miR-302/367 cluster in astrocytes increased the endogenous potential for repairing myelin insults by the generation of oligodendroglia from astrocytes	[140]
Wang et al.	2017	miR-219	miRNA mutant mice, siRNA transfection in primary OPCs, mimic delivery in vivo injected into white matter of 6- to-8-week-old WT mice containing 1% LPC	(1) Deletion of miR-219 genes leads to OL differentiation defects in the developing CNS and impaired CNS myelination; (2) miR-219 overexpression promotes OL maturation and remyelination; (3) miR-219 targets stage-specific inhibitors to promote OL differentiation; (4) miR-219 mimic promotes remyelination after LPC-induced demyelinating damage	[141]
Zhang et al.	2017	miR-146a	Infusion of mimics into the corpus callosum for 7 days	(1) miR-146a mimics facilitated remyelination associated with augmentation of newly generated mature OLs; (2) miR-146a treatment considerably reduced IRAK1 protein levels	[142]
Fan et al.	2017	miR-219	Transduction of OPCs with miR-219-GV254 lentivirus and co-culture with spinal cord explant and transplantation in CPZ-induced demyelination model	(1) miR-219 accelerated the differentiation of NPCs into OPCs; (2) after transplantation of miR219-OPCs into CPZ-induced demyelinated mice, OLs migrated and matured to express MBP; (3) the presence of new myelin sheaths was observed; (4) transplanted miR219-OPCs induced the proliferation of endogenous NPCs	[143]
Liu et al.	2017	miR-219	Administration of miR-219-GV254 lentivirus into the corpus callosum of CPZ-induced demyelination mice	(1) miR-219 decreased the quantity of OPCs; (2) miR-219 increased the number of OLs and the levels of MBP and CNP; (3) miR-219 overexpression attenuated the extent of demyelination	[144]
Kuypers et al.	2016	miR-297c	miR-297c lentivirus transduction in mouse embryonic fibroblasts and rat OPCs	(1) Overexpression of miR-297c-5p promoted G_1_/G_0_ arrest; (2) miR-297c-5p transduction increased the number of O1^+^ rOPCs during differentiation; (3) miR-297c-5p targeted CCNT2	[145]
Lecca et al.	2016	miR-125a-3p	Mimic/AntagomiR in primary OPCs from rat	(1) miR-125a-3p over-expression impaired OL maturation; (2) miR-125a-3p inhibition stimulated OL maturation	[130]
Ebrahimi-Barough et al.	2013	miR-219	miR-219-GFP-expressing lentivirus in hOPCs	(1) Overexpression of miR-219 decreased PDGFRa mRNA in OPCs and promoted pre-OL differentiation fate	[147]

Table 2. Summary of studies with functional therapeutic evidence of miRNAs in MS repair. In addition to the main author, year of publication and corresponding reference, the table includes information on the miRNA of interest and its therapeutic mode of administration. The major findings of each study regarding remyelination and neurorepair are also summarized. OLs: oligodendrocytes; LPC: lysolecithin; OPCs: OL precursor cells; EAE: experimental autoimmune encephalomyelitis; CPZ: cuprizone; P1–2; postnatal 1–2; GFP: green fluorescent protein; hiPSCs: human induced pluripotent stem cells; siRNA: small interfering RNA; NPCs: neural progenitor cells; MBP: myelin basic protein; CNP: 2′,3′-Cyclic nucleotide 3′-phosphodiesterase; hOPCs: human OPCs.

**Table 3:** Studies with the generation of human MS models.

Study	Year	Type of MS	Starting Cell Type	Method of Reprogramming	Induced Cell Type	Major Findings	Reference
Fagiani et al.	2024	RRMS	Human fibroblasts	Sendai virus	Organoids containing microglia	(1) Accelerated OL differentiation from hiPSC-derived NPCs to generate forebrain organoids; (2) inclusion of hiPSC-derived microglia to achieve immunocompetence; (3) mimicking macroglia–microglia neurodegenerative phenotypes in organoids after being exposed to inflamed CSF from pwMS	[195]
Marotta et al.	2024	PPMS	Biopsy-derived fibroblast	mRNA	Organoids containing isogenic microglia	(1) Organoids cultured in LEO had lower levels of genes associated with cell proliferation and higher levels of maturation-associated genes	[196]
Ionescu et al.	2024	PMS	Skin fibroblasts	Non-modified RNA plus microRNA	iNSCs	(1) PMS iNSCs leaded to neurotoxic signaling, which might be pharmacologically rescuable	[197]
Clayton et al.	2024	RRMS + SPMS + PPMS	Skin biopsies	Modified mRNAs	Glia-enriched cultures, cortical neurons	(1) Fewer OLs in PPMS iPSC-derived cultures; (2) increased expression of immune and inflammatory genes in OL lineage cells and astrocytes	[24]
Gisevius et al.	2024	MS	RPTEC	Episomal plasmids	Primary neurons	(1) Promoted neurite recovery by propionic acid in induced primary neurons	[198]
Daviaud et al.	2023	RRMS + SPMS + PPMS	PBMCs	Episomal plasmids	Cerebral organoids	(1) In MS organoids: (a) disrupted stem cell proliferation capacity, (b) reduced stem cell pool, (c) increased NPC and neuronal population and reduced OL population, (d) reduced expression of cell cycle inhibitor p21, (e) senescent and prematurely differentiated NPCs	[199]
Kerkering et al.	2023	RRMS + SPMS + PPMS	PBMCs	Sendai virus	NSCs, neurons, and astrocytes	(1) Increased neurite damage in MS neurons treated with TNF-α/IL-17A; (2) less axonal damage in HC neurons cultured with TNF-α/IL-17A–reactive BMS astrocytes than PMS astrocytes; (3) rescued TNF-α/IL-17–induced neurite damage with supernatants from BMS astrocyte/neuronal cocultures	[200]
Lotila et al.	2022	MS	PBMCs	Sendai virus		(1) Establishment of iPSC line	[201]
Begentas et al.	2022	MS	PBMCs	Sendai virus	iPSCs	(1) Establishment of iPSC line	[202]
Fortune et al.	2022	RRMS + SPMS	PBMCs	Sendai virus	iPSCs	(1) Generation of 4 MS iPSC lines within a single family	[203]
Nishihara et al.	2022	RRMS	PBMCs	Episomal plasmids	BMECs	(1) In MS-derived BMEC-like cells: (a) impaired junctional integrity, barrier properties and efflux pump activity, (b) inflammatory phenotype with increased adhesion molecule expression and immune cell interactions, (c) enhanced barrier characteristics and reduced inflammatory phenotype when Wnt/β-catenin signaling is activated	[204]
Ghirotto et al.	2022	RRMS	PBMCs	Episomal plasmids	Astrocytes	(1) In MS astrocytes, including (a) enriched expression of genes associated with neurodegeneration, (b) increased mitochondrial fission, (c) increased production of superoxide and MS-related proinflammatory chemokines, (d) altered glutamate uptake/release, (e) increased electron transport capacity and proton leak, (f) distinct metabolic profile	[205]
Smith et al.	2022	MS	PBMCs	Sendai virus	Astrocytes	(1) No differences between MS and control stimulated astrocytes; (2) suppressed OPC differentiation and myelin gene expression in OPCs exposed to ACM; (3) induction of immune pathways in OPCs exposed to ACM	[206]
Plastini et al.	2022	PPMS	Skin biopsies	mRNA/miRNA method	OLs	(1) Differentially expressed genes in PPMS OLs (cell adhesion, apoptosis and inflammation)	[207]
Begentas et al.	2021	RRMS	PBMCs	Sendai virus	iPSCs	(1) Establishment of iPSC line	[208]
Mehta et al.	2021	PPMS	PBMCs	Sendai virus	iPSCs	(1) Establishment of iPSC line	[209]
Mutukula et al.	2021	RRMS + PPMS	PBMCs	Episomal plasmids	iPSCs + NPCs	(1) Elevated expression of senescence hallmarks	[210]
Starost et al.	2020	RRMS	Skin biopsies	Sendai virus	OLs, neurons	(1) No differences between RRMS and control OLs in (a) proliferation and migration capacities, (b) differentiation, (c) ensheathment of 3D nanofibers, (d) stress response, (e) proteome. (2) Impaired differentiation of both OLs with supernatants of supernatants of activated PBMCs	[211]
Lopez-Caraballo et al.	2020	SPMS	Menstrual blood-derived stromal cells	DNA vectors	OLs	(1) Differences between SPMS-OPCs and control-OPCs in (a) transcription products; (b) protein secretion into CM. (2) Deficient capacity to stimulate OPC migration in SPMS-OPC CM.	[212]
Morales Pantoja et al.	2020	PMS	PBMCs	Episomal plasmids	OLs	(1) Exposure to IFNγ might (a) inhibit OPC differentiation, (b) redirect lineage phenotype, (c) upregulate immune response pathways	[213]
Perriot et al.	2018	RRMS	PBMCs	Episomal plasmids	Neurons, Astrocytes	(1) Relevant use of serum-free media to generate resting astrocytes; (2) specific reactivity of astrocytes depending on the triggering inflammatory stimulus	[214]
Miquel-Serra et al.	2017	MS	Skin fibroblasts	Retroviral transduction	iPSCs	(1) Establishment of iPSC line	[215]
Nicaise et al.	2017	PPMS	Blood	Sendai virus	NPCs	(1) PPMS NPCs failed to protect against (a) CPZ-induce demyelination, (b) toxicity; (2) did not support OL differentiation by PPMS NPCs	[216]
Massa et al.	2016	MS	RPTEC	Episomal plasmids	Primary neurons	(1) Establishment of iPSC line with a non-invasive procedure devoid of permanent genetic manipulation	[217]
Douvaras et al.	2014	PPMS	Skin biopsies	mRNA/miRNA method	OLs	(1) Functional PPMS OPCs with myelination capacity	[218]
Herszfeld et al.	2014	MS	Fibroblasts	Retroviral transduction	OLs	(1) Obtention of mixed population of neural cells rather than oligodendroglia of high purity	[219]
Song et al.	2012	RRMS	Fibroblasts	Retroviral transduction	iPSCs	(1) Establishment of iPSC line from a MS patient	[194]

MS: multiple sclerosis; RRMS: relapsing–remitting MS; OLs: oligodendrocytes; iPSCs: induced pluripotent stem cells; hiPSCs: human iPSCs; NPCs: neural progenitor cells; CSF: cerebrospinal fluid; pwMS: people with MS; PPMS: primary progressive MS; mRNA: messenger RNA; LEO: low Earth orbit; PMS: progressive MS; iNSCs: induced neural stem cells; SPMS: secondary progressive MS; RPTEC: renal proximal tubule epithelial cells; PBMCs: peripheral blood mononuclear cells; BMS: benign MS; BMECs: brain microvascular endotelial cells; OPCs: OL precursor cells; ACM: astrocyte conditioned media; miRNA: microRNA; CM: conditioned media; CPZ: cuprizone.

## Abbreviations

The following abbreviations are used in this manuscript:

2D	Bidimensional
3D	Tridimensional
AAV	Adeno-associated virus
AMOs	Anti-miRNA oligonucleotides
ASOs	Antisense oligonucleotides
BBB	Blood brain barrier
BMECs	Brain microvascular endothelial cells
BoC	Brain-on-a-chip
CNS	Central nervous system
CPZ	Cuprizone
CSF	Cerebrospinal fluid
EAE	Experimental autoimmune encephalomyelitis
EB	Ethidium bromide
EVs	Extracellular vesicles
HBSs	Human brain slices
hfNPCs	Human fetal neural progenitor cells
iPSCs	Induced pluripotent stem cells
LPC	Lysolecithin
LV	Lentivirus
MBP	Myelin basic protein
miRNAs	microRNAs
MRI	Magnetic resonance imaging
MS	Multiple sclerosis
MSCs	Mesenchymal stem cells
NPCs	Neural progenitor cells
NSCs	Neural stem cells
OBSs	Organotypic brain slices
OLs	Oligodendrocytes
OoAC	Organs-on-a-chip
OPCs	Oligodendrocyte progenitor cells
PMS	Progressive forms of MS
RRMS	Relapsing–remitting MS
shRNAs	Short hairpin RNAs
TSBs	Target-site blockers
